# Intact embodiment during perspective-taking in older adults is not affected by focal tDCS

**DOI:** 10.1007/s11357-025-01554-4

**Published:** 2025-02-19

**Authors:** Mandy Roheger, Anna Mäder, Steffen Riemann, Filip Niemann, Klaus Kessler, Andrew K. Martin, Marcus Meinzer

**Affiliations:** 1 Department of Psychology, Carl von Ossietzky Universität Oldenburg, Oldenburg, Germany; 2 Department of Neurology, University Medicine Greifswald, Greifswald, Germany; 3 School of Psychology, University College Dublin, Dublin, Ireland; 4 Kent and Medway Medical School, Canterbury, UK; 5 School of Psychology, University of Kent, Canterbury, UK

**Keywords:** Social cognition, Aging, TDCS, RTPJ, DmPFC, Visual perspective tracking, Visual perspective taking, Current modelling

## Abstract

Embodied processing is crucial for visual perspective taking (VPT), with evidence from non-invasive transcranial direct current stimulation (tDCS) suggesting a causal role of the right temporoparietal junction (rTPJ). However, it is not known whether such embodied factors are maintained in older adults or whether rTPJ-tDCS has comparable effects in advanced age. We employed a balanced and sham-tDCS controlled, double-blinded, cross-over design, including two randomized experimental groups of healthy older adults, receiving focal tDCS over either the rTPJ (*n* = 30), or a control region in the dorsomedial prefrontal cortex (dmPFC, *n* = 30). A healthy young control group (*n* = 30, not receiving tDCS) was included to investigate potential changes in embodied processing in older adults. All groups completed neuropsychological baseline testing and an experimental VPT paradigm, in which perspective-taking (requiring embodied rotation) and perspective-tracking (line-of-sight judgements) were assessed. Structural magnetic resonance imaging data were acquired to conduct individualized current flow simulations, aimed at identifying potential changes in neurophysiological effects of tDCS in older adults. Older adults responded slower across perspective tracking and perspective taking tasks but showed comparable embodied effects of body posture and angle of rotation during perspective taking. Contrary to previous results in younger adults that demonstrated regionally and task-specific effects of focal rTPJ-tDCS, no stimulation effects on embodied processing were found in older adults. Electrical field simulations suggested focal current delivery in both age-groups but also significantly reduced current strength in the target regions for tDCS in older adults. Older adults are as embodied as young adults during perspective taking. However, tDCS administered to the rTPJ or dmPFC had no effect, which may be explained by reduced current delivery to the target regions due to age-associated changes in skull and brain anatomy and/or functional brain reorganization. Our results are in line with previous studies suggesting that tDCS effects obtained in young participants may not translate directly to advanced age. Future studies could address this by using individualized modelling approaches aimed at adjusting current dose for (older) study participants and pre-stimulation functional imaging involving VPT tasks-of-interest, to identify optimized target regions for tDCS.

Registration: ClinicalTrials.gov Identifier: NCT04633499.

## Introduction

The right temporoparietal junction (TPJ) is a critical brain region located at the intersection of the parietal and temporal lobes, often described as a key hub of the social brain [1], which is thought to play a central role in understanding social interactions and others' mental states**.** This brain region has been associated with several specific socio-cognitive processes, such as perspective-taking and theory of mind, which are relevant to social functioning [2]. One such process centers on the theory of embodiment, referring to how cognition emerges through an interaction between brain and body, rather than simply being a mental process siloed within the brain. For example, when we try to understand how someone else views the world, we often mentally simulate their perspective by performing an embodied rotation. This involves mentally aligning our own viewpoint to that of the other person [3]. In other words, we place ourselves metaphorically “in their shoes”. This embodied social cognitive process has consistently been associated with the right TPJ [4–6]. Moreover, because both physical (e.g., height decreases, postural change, decreased motor speed) and mental changes [7] are frequent in advanced age [8], embodiment has also been put forward as a key theoretical consideration for understanding age-related changes in socio-cognitive processing [9, 10]. Notably, if the physical and cognitive manifestations of ageing are intertwined, especially for embodied processes such as performing a rotation of one’s visual perspective to that of another person, then older adults’ performance on embodied tasks should disproportionately be impaired. Moreover, it has been suggested that older adults become less embodied with age, relying more on visual processing over bodily factors [11], suggesting that alternate neural mechanisms may be employed to perform tasks requiring embodied processing. However, despite theoretical positions on embodiment and ageing, little empirical research has been conducted in this field.

Visual perspective taking (VPT) tasks are particularly suited to investigate these issues, because they can be divided into embodied and non-embodied components. For example, understanding how someone imagines a scene is thought to rely on embodied processes and is often labelled as Level 2 perspective taking. In contrast, simply understanding what another person can see from their viewpoint requires only a basic line-of-sight judgment without the need for embodied rotation. This is referred to as Level 1 perspective taking or perspective tracking task [3]. The contrasting strategies for performing such tasks are highlighted in several studies [3–5, 12] using a version of a cognitive task where an avatar was placed at one of three angular disparities to the perspective of the participant. Participants were required to answer whether an object was visible to the avatar or whether it was visible on the avatar’s left or right. The angular disparity only affected response times when participants had to determine whether the object was on the avatar’s left or right side. Greater angular disparity was associated with slower responding, thus demonstrating an embodied rotation strategy. Additionally, participants were sat in a position either aligned (congruent) or misaligned (incongruent) with the positioning of the avatar, resulting in slower responding when body position was incongruent, but only for laterality judgements and not simple line-of-sight visibility judgements. The task is therefore ideal for identifying embodied processes in older adults, especially those relevant to social cognition. If older adults are less embodied then they should not show the same angular disparity or body position effects as those observed in younger adults. However, if embodiment is not directly and causally linked to action efficiency it may persist as a learned cognitive mechanism despite bodily deterioration. Here, we would show comparable embodied processes during perspective taking in older adults as those observed in young adults.

Two key brain regions associated with VPT are the temporoparietal junction (TPJ) [13, 14] and the dorsomedial prefrontal cortex (dmPFC) [13, 15]. Even though results from studies using non-invasive brain stimulation that allow modulating neural excitability to establish the causal role of specific brain regions in a given task have been mixed [16], the right temporoparietal junction is often linked with embodied processing, relevant for perspective taking. For example, a study by [5] that used focal transcranial direct current stimulation (tDCS) during a VPT paradigm in younger adults showed that excitatory “anodal” stimulation of the rTPJ increased the effect of body posture congruency (congruence with the direction of mental self-rotation) during perspective-taking, whereas no such effects were identified during perspective-tracking, or for dmPFC stimulation.

Yet, most non-invasive brain stimulation studies focused on investigating VPT are solely in young adults. As socio-cognitive skills and also VPT abilities decrease in older age [10], it is important not only to investigate the contribution of embodied factors to VPT in advanced age but also to assess if anodal tDCS stimulation of the rTPJ is effective in older adults to selectively improve perspective-taking. So far, research on VPT and the influence of rTPJ and dmPFC tDCS on VPT performance in older adults is scarce [6, 17] and showed age-specific slowing in the response reaction times compared to younger adults, but no specific stimulation effect. Hence, the motivation of the current study are twofold: to assess whether older adults are less embodied when required to understand the perspective of others and to investigate whether the causal relationship between embodied VPT and the right TPJ, previously confirmed by non-invasive brain stimulation in young adults, is also observed in older adults. We also explored if potential differences in tDCS response of older adults can be explained by changes in current flow to the target regions for tDCS by using individualized current flow modelling.

## Materials and methods

The present study was pre-registered (Identifier: NCT04633499, clinicaltrials.gov). Ethical clearance was granted by the University Medicine Greifswald (No. BB 100/20). Reporting followed the CONSORT guidelines for reporting randomized trials [18].

### Participants

Thirty healthy young adults (17 females, mean age: 22.9 [2.32]) and 61 healthy older adults (37 females, mean age: 69.1 [3.97]) were recruited through online and offline advertisement, and the study that was conducted at the University Medicine of Greifswald. The group of young adults only participated in baseline testing and one experimental session without additional brain stimulation, whereas the group of older adults was randomized to receive either dmPFC (*n* = 30, 17 females, mean age: 69.1 [3.84]) or rTPJ (*n* = 31, 20 females, mean age: 69.2 [4.16]) anodal tDCS in a sham-controlled, double-blind, randomized cross-over study. Participants were native German speakers with corrected to normal vision and hearing. To be included in the present study, younger adults needed to be between 18 and 35 years of age and older adults between 60 to 80 years. Participants were not currently taking psychoactive medications or substances and had no history of neurological or mental illness. Written informed consent was obtained from each participant prior to the first experimental session, and participants were compensated for their time with a small monetary amount. Furthermore, older participants completed a safety screening questionnaire before the experimental brain stimulation.

### Study design, randomization, and allocation

The present study is a sham-controlled, double-blind, cross-over study including two randomized experimental groups (healthy older adults, either dmPFC or rTPJ tDCS stimulation) and a healthy young control group. All participants received baseline testing and completed structural MRI scans for individualized current modelling. The healthy young adults then participated in a single experimental session in which they conducted the Reading-The-Mind-In-The-Eyes of Children Task (RME-C-T [19]), as well as a task on visual perspective taking [3, 5]. Healthy older participants were randomized to receive tDCS over the dmPFC or the rTPJ.

Randomization was achieved using a random number generator to allocate healthy older participants to the two groups in a 1:1 allocation (simple randomization approach) and was prepared by an investigator not involved in the experimental procedures. Furthermore, stimulation order was balanced across both groups, meaning that half of the participants received active and half received sham stimulation during their first experimental session, again using a computer-generated randomization list. After the principle investigators had obtained the participant’s consent, they contacted an investigator who was independent of the recruitment process for allocation consignment.

### Baseline testing

At baseline testing, all participants were asked to complete cognitive assessments to ensure that the two experimental groups (healthy older people: dmPFC vs. rTPJ stimulation sites) were comparable and that all participants scored within age-corrected norms. We acquired information on demographic variables, the Edinburgh Inventory for Handedness [20], and the cognitive assessment battery CERAD-Plus (https://www.memoryclinic.ch/de/main-navigation/neuropsychologen/cerad-plus/), which comprises tests for assessing verbal fluency (naming as many animals as possible in one minute), the Boston Naming Test (BNT [21]), the German version of the Mini-Mental Status Examination (MMSE [22]), a test for verbal and figural short- and long-term memory (a word list consisting of 15 words, and a test containing six different geometrical figures), the trail making task A and B (TMT A and B [23]) and a phonemic fluency task (naming as many words starting with the letter S as possible in one minute).

### Structural MRI

MRI data were acquired with a 3-T Siemens Verio scanner at the University Medicine Greifswald using a 32-channel head coil. For current flow modeling, we obtained T1- (1 mm^3^ isotropic voxels, TR = 2300 ms, TE = 2.96 ms, TI = 900 ms, flip angle 9°) and T2-weighted images (1 mm^3^ isotropic voxels, TR = 12770 ms, TE = 86 ms, flip angle 111°) of each participant. Data were acquired prior to the experimental sessions, and no electrodes were attached.

### tDCS

We used a focalized tDCS set-up [5], which provides greater regional precision of current delivery compared to conventional (pad-based) tDCS. Anodal stimulation was delivered using a one-channel direct current stimulator DC-Stimulator Plus (NeuroConn). The anode was a small circular rubber electrode (2.5 cm in diameter); the return electrode was ring-shaped and placed equidistantly around the anode. As in our previous study in healthy young adults [5], we used two different electrode sizes for the two stimulation sites: At the rTPJ, the return electrode was slightly smaller than at the dmPFC (rTPJ: inner/outer diameter: 7.5/9 cm; dmPFC: inner/outer diameter: 9.2/11.5 cm). The smaller size of the rTPJ set-up was necessary to avoid overlap of the cathode with the right ear. Electroconductive gel (Weaver Ten20 conductive paste) and an EEG cap ensured consistent adhesion of the electrodes to the skin. Using the 10–20 EEG system as a reference for our electrode placement, the dmPFC was located 15% of the distance from FZ towards FPz, and the rTPJ was located at CP6. At both stimulation sites and for both sham and active stimulation, the current ramped up to 1 mA over 8 s and ramped down over 5 s. In the “sham” condition, the current was maintained at 1 mA for 40 s, whereas in the active condition, the current was maintained at 1 mA for 20 min. The procedures during the VPT task were identical as described in Martin et al. (2020), including that another experimental task preceded the VPT experiment (RMET). Hence, the stimulation was already running for approximately 10–12 min, depending on the reaction speed of the participants, when the VPT started. This also means that the majority of the VPT task relied on physiological after-effects of tDCS (offline effects), that are known to extend beyond the actual current delivery [24]. Notably, a previous study from our group demonstrated that there were no differences in cognitive tDCS effects between online and offline effects [25] and Martin et al. (2020) demonstrated regionally and task specific tDCS effects in younger adults using the same stimulation design. Researchers were blinded to the stimulation condition using the “study-mode” of the DC-Stimulator (i.e., a pre-assigned code triggered either anodal or sham tDCS). Participants were also blinded to the stimulation conditions. To avoid carryover effects from active to sham tDCS conditions, experimental cross-over sessions were scheduled at least one week apart.

### Visual-perspective task

**Fig. 1:**
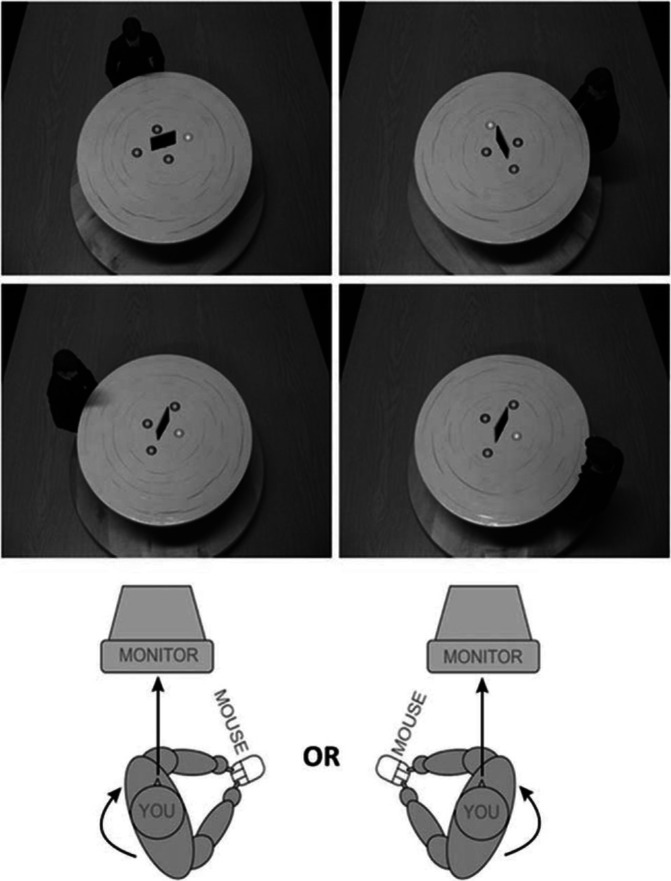
Experimental setup

We assessed perspective-tracking (VPT Level 1) and visual perspective-taking (VPT Level2) using a VPT tracking/taking task by [26], modified as described by [5]. In this task, a table was presented on a computer monitor on which an avatar sat on one of six locations (at 60°, 110°, or 160° from the left or right of the gaze of the participant who was seated in front of a computer screen, see also Fig. 1). The different angles were included as a manipulation on how far the participant in front of the monitor would have to mentally rotate (themselves) to transform their perspective onto the perspective of the avatar. On the table, four grey discs were arranged around an occluding panel. On each trial, one of the four discs would be illuminated in red to indicate the target. In the perspective-tracking condition (VPT 1), participants were asked whether the disc was visible to the avatar (Yes or No response, conducted with left or right button press), whereas in the perspective-taking condition (VPT 2), participants were asked whether the disc was on the avatar’s left or right (left or right response, conducted with left or right button press). The participant’s body position was manipulated to be either congruent or incongruent to the positioning of the avatar around the table in order to manipulate embodied processing. Previous research had demonstrated that a posture that was congruent with the direction of mental self-rotation (into the avatar’s perspective) significantly speeded up left/right judgements showing mental embodiment of the avatar’s orientation [3, 26]. Only left/right judgements (VPT-2) but not visibility judgements (VPT-1) revealed this effect [3]. To reliably manipulate posture, we marked the positions on the floor and asked the participants to swivel the chair to the marked position. Participants were instructed to not respond until their chair and body was stationary. Both perspective-tracking and -taking were presented in 14 alternating blocks of 24 trials each. Furthermore, 12 practice trials (six for each condition) were administered at the beginning of the session to ensure participants understood task instructions.

### Adverse effects

Possible adverse events were assessed at the end of each stimulation session using a short questionnaire consisting of seven possible symptoms that may have occurred during stimulation: itching, pain, burning, heat, metallic taste, reduced attention, and other symptoms [27]. Furthermore, mood was assessed using the Positive and Negative Affect Scale (PANAS, [28]).

### Statistical analyses

All analyses were conducted in SPSS, version 25 (IBM). Analyses were conducted similar to the original study of [5]. For the descriptive statistics, continuous variables were summarized as means and standard deviations or as medians and interquartile ranges, as appropriate. For categorical variables, data were presented as number of cases and percentages. To calculate *p*-values for differences, ANOVAs were used for continuous and chi-square tests for categorical variables. Repeated-measures ANOVAs were computed for both perspective-tracking and-taking conditions for the groups of older adults receiving stimulation. The outcome was response time (for correct answers only), and the predictors were stimulation type (STIM TYPE; sham/anodal), stimulation site (STIM SITE; dmPFC/rTPJ), body position (POSITION; congruent/incongruent), and angle of rotation (ANGLE; 60°, 110°, 160°), with STIM SITE as a between-subjects factor. Where violations of sphericity were detected, Greenhouse–Geisser corrections were used. To detect differences between younger and older adults without stimulation in our paradigm, we conducted two 2 × 2 × 3 repeated-measures ANOVA with response time as the outcome and age group, body position, and angle of rotation as predictors. As congruency effects can be masked or amplified by slower response times, we also include processing speed as a covariate, calculated as the average response time across all angles for the congruent condition. This allows us to calculate age-related differences on congruency effects in Level 2 perspective taking independent of age-related slowing in processing speed.

### Electric field simulations

Current flow simulations were conducted using SimNIBS 4.0 [29, 30]. Individualized tetrahedral head meshes were generated with *charm,* based on individual T1- and T2-weighted images of each participant to estimate current flow patterns for the respective tDCS montages targeting either the dmPFC or rTPJ [31]. Freeview (FreeSurfer; https://surfer.nmr.mgh.harvard.edu/) was used for inspection of the reconstructed tissue compartments, and five participants had to be excluded due to head reconstruction failure (young *N* = 2; old dmPFC/rTPJ *N* = 1/2).

Simulation parameters were chosen to reflect the experimental setup of this study (i.e., intended positions of the center anode and ring cathode, electrode dimensions, current strength, gel thickness). Electrode thickness and gel thickness were defined as 2 and 1 mm, respectively. Standard conductivity values as provided by SimNIBS were used.

For the dmPFC montage, the center position of the anode was initially determined in MNI space by identifying FPz and Fz and measuring the distance between the two points. The target scalp coordinates were then determined by measuring 15% of the distance from Fz towards FPz (MNI scalp coordinates x/y/z 0.5/71.7/46.1). The center position of anode for the rTPJ montage corresponded to the CP6 position of the EEG-10–10 system provided by SimNIBS. Ring cathodes were placed around the center anodes with equal spacing. Subsequently, all electrodes were transformed into subject space for individualized modeling.

Individualized simulations for older participants reflected the stimulation protocol they had received (i.e., dmPFC or rTPJ tDCS). For the young age group, both sites were simulated to show the theoretical current flow in the target sites (because this group did not receive stimulation during the experiment). Current flow was simulated in subject space. For illustrative purposes, weighted group averages of the electric field strength for each age-group and montage were calculated in FsAverage (i.e., the standard surface-based coordinate system as distributed with Freesurfer) space.

For direct comparison of current flow to the target regions for tDCS between young and older participants, the cortical electric field (magnitude E) was extracted at the intersection of spherical regions of interest (ROIs) and the cortical grey matter underneath the center anodes. ROIs with a radius 12.5 and 25 mm were chosen to illustrate current flow to the immediate target region (i.e., 12.5 mm ROI) and the focality of the stimulation; i.e., the comparison of current flow to the larger vs. smaller ROI (for details see [32]). The centers of the respective ROIs were positioned at MNI *x*/*y*/*z* coordinates 0/54/33 (dmPFC) and 60/ − 54/13 (rTPJ), which showed peak electric field strength in a previous study using the same montages [33]. Current strength in the target regions was compared using ANOVAs with the factors age group (young, old) and ROI radius (12.5 mm, 25 mm) to investigate focality. ANOVAs were computed individually for dmPFC and rTPJ to avoid unbalanced group sizes.

## Results

### Participant demographics

**Fig. 2:**
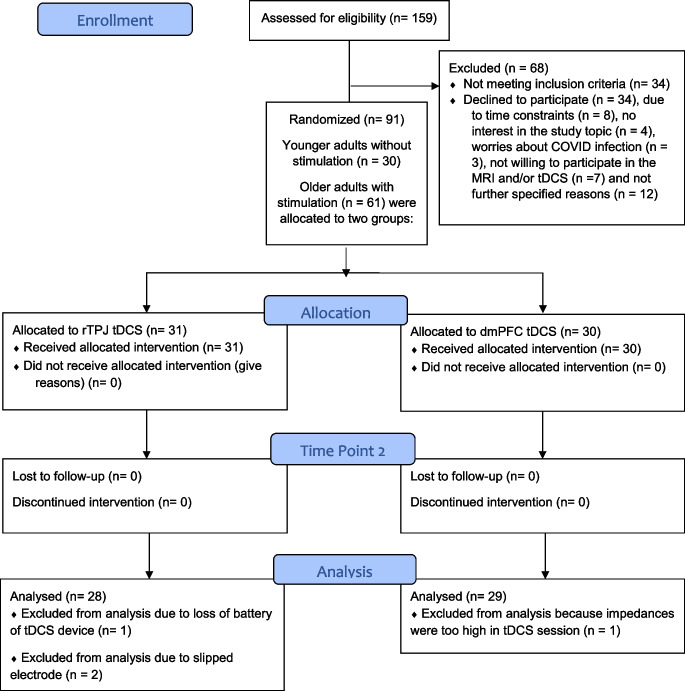
Participant Flow throughout the study

A flow diagram of the participant flow with reasons for inclusion and exclusion is provided in Fig. 2. We assessed 159 participants for eligibility and had to exclude *n* = 34 participants as they were not meeting inclusion criteria. Another *n* = 34 participants did not want to participant due to time constraints (*n* = 8), no interest in the study topic (*n* = 4), fear of getting a Covid-19 infection (as we recruited during the pandemic in 2020–2022; *n* = 3), fear of conducting an MRI (*n* = 6) or tDCS testing (*n* = 1), and reasons not specified (*n* = 12). Participants’ demographic data (including age, sex, education) and their test scores in the neuropsychological testing are presented in Table 1. Importantly, there were no significant differences in the demographics and the neuropsychological testing data between the two older groups (i.e., rTPJ vs. dmPFC stimulation). As expected, neuropsychological test performance differed significantly between younger and older adults in all tests (young > older), except for performance on the Boston Naming Test and the Figure Copy Task. Older and younger adults did not differ regarding years of education.

**Table 1:** Participants’ demographics and neuropsychological testing scores

	Young adults (*n* = 30)	Older adults Overall (*n* = 57)	Difference between young and old adults	Older adults rTPJ group (*n* = 28)	Older adults dmPFC group (*n* = 29)	Difference between rTPJ and dmPFC
	M (SD)	*M* (SD)	*p*-value	*M* (SD)	*M* (SD)	*p*-value
Demographics						
Age	22.9 (2.32)	69.3 (3.84)	** < .05**	69.5 (3.82)	69.1 (3.91)	0.650
Sex	17 ♀ 13 ♂	33 ♀ 24 ♂	0.915	17 ♀ 11 ♂	16 ♀ 13 ♂	0.672
Education (in years)	15.8 (1.80)	16.1 (2.35)	0.491	16.3 (2.54)	16.0 (2.20)	0.651
Handedness (*n* right)	24	50	0.589	26	24	0.261
Neuropsychological testing						
MMSE (max. 30 points)	29.8 (0.77)	29.2 (0.87)	** < .01**	29.2 (0.79)	29.1 (0.95)	0.743
BNT (max. 15 points)	14.9 (0.25)	14.9 (0.31)	0.559	14.9 (0.32)	14.9 (0.31)	0.965
Semantic fluency	26.2 (4.87)	23.2 (5.94)	** < .01**	24.6 (5.30)	21.8 (6.25)	0.066
Phonematic fluency	18.1 (4.05)	15.9 (4.15)	** < .05**	16.1 (4.18)	15.2 (4.18)	0.731
WL—immediate recall (max. 10 points)	6.6 (1.59)	5.63 (1.74)	** < .001**	5.5 (1.77)	5.7 (1.73)	0.686
WL—delayed recall (max. 10 points)	8.83 (1.26)	6.95 (1.57)	** < .01**	6.9 (1.58)	7.0 (1.58)	0.793
WL—recognition (max. 10 points)	10.0(0.0)	9.7(0.64)	** < .01**	9.6 (0.69)	9.7 (0.59)	0.493
Figure copy (max. 11 points)	10.6(0.73)	10.4(0.73)	0.439	10.5(0.87)	10.4 (0.57)	0.920
Figure recall (max. 11 points)	10.2 (1.19)	9.4 (1.44)	** < .05**	9.4(1.47)	9.6 (1.43)	0.495
TMT A (in seconds)	24.9 (6.9)	41.8 (12.9)	** < .001**	44.4(15.20)	39.2 (9.90)	0.135
TMT B (in seconds)	46.0 (12.0)	83.4 (30.0)	** < .001**	88.4 (36.2)	78.6(22.1)	0.222

Please note that neuropsychological testing scores are reported as raw scores, as the CERAD testing battery does not provide norms for younger adults. Bold values indicate a significant difference

*Abbreviations*: *M* mean, *SD* standard deviation, *rTPJ* right temporoparietal junction, *dmPFC* dorsomedial prefrontal cortex, *MMSE* Mini Mental Status Examination, *BNT* Boston Naming Test, *WL* Wordlist, *TMT* Trial Making Test

### Perspective-tracking and -taking

All perspective-tracking and -taking response times across all conditions are presented in Table 2.

**Table 2:** Response times in millisecond (reported as means and standard deviations) for perspective-tracking (Level 1) and perspective-taking (Level 2) during sham and anodal HD-tDCS at the rTPJ and dmPFC in older adults and without stimulation in younger adults

	rTPJ		dmPFC		Young adults
	Sham	Anodal	Sham	Anodal	
Perspective-tracking					
Congruent					
60°	1169 (296)	1122 (235)	1084 (243)	1057 (243)	787 (187)
110°	1186 (341)	1092 (195)	1063 (228)	1060 (261)	766 (196)
160°	1172 (392)	1143 (246)	1050 (245)	1077 (301)	764 (243)
Incongruent					
60°	1195 (358)	1169 (325)	1059 (198)	1075 (267)	788 (246)
110°	1166 (385)	1117 (226)	1040 (212)	1045 (252)	770 (204)
160°	1157 (322)	1070 (213)	1041 (221)	1052 (287)	744 (186)
Perspective-taking					
Congruent					
60°	1240 (403)	1202 (340)	1117 (357)	1190 (477)	703 (228)
110°	1242 (426)	1202 (331)	1117 (382)	1256 (527)	752 (235)
160°	1411 (445)	1326 (305)	1339 (541)	1399 (583)	878 (270)
Incongruent					
60°	1370 (299)	1302 (313)	1185 (411)	1282 (574)	711 (399)
110°	1389 (381)	1340 (345)	1272 (544)	1366 (574)	815 (281)
160°	1539 (407)	1510 (501)	1388 (469)	1487 (624)	921 (307)

#### Differences in perspective-tracking (Level 1 VPT) and taking (Level 2 VPT) between younger and older adults.

##### Perspective tracking (VPT1)

Overall, older adults responded slower than younger individuals, *F*(1,55) = 48.06, *p* < 0.001, *η*^2^_*p*_ = 0.47. No main effects were identified for angle, *F*(2,110) = 2.61, *p* = 0.08, *η*^2^_*p*_ = 0.05 or posture, *F*(1,55) = 1.10, *p* = 0.30, *η*^2^_*p*_ = 0.02. Age group did not interact with angle, *F*(2,110) = 0.05, *p* = 0.96, *η*^2^_*p*_ < 0.001 or with posture, *F*(1,55) = 0.33, *p* = 0.57, *η*^2^_*p*_ = 0.01. There was no interaction between angle and posture, *F*(2,110) = 0.75, *p* = 0.47, *η*^2^_*p*_ = 0.01, and the three-way interaction with age group was not significant, *F*(2,110) = 0.37, *p* = 0.69, *η*^2^_*p*_ = 0.01.

##### Perspective taking (VPT2)

Older adults responded slower in general, *F*(1,54) = 33.60, *p* < 0.001, *η*^2^_*p*_ = 0.38. There was a significant main effect of angle, *F*(2,108) = 50.16, *p* < 0.001, *η*^2^_*p*_ = 0.48 and posture, *F*(1,54) = 37.85, *p* < 0.001, *η*^2^_*p*_ = 0.41. Age group did not interact with posture, *F*(54) = 2.63, *p* = 0.11, *η*^2^_*p*_ = 0.05 or angle; *F*(2,108) = 1.97, *p* = 0.14, *η*^2^_*p*_ = 0.04. There was no interaction between angle and posture, *F*(2,108) = 0.54, *p* = 0.58, *η*^2^_*p*_ = 0.01, and no three-way interaction with age group, *F*(2,108) = 0.56, *p* = 0.57, *η*^2^_*p*_ = 0.01.

Because slower responses can mask or amplify congruency effects, we also conducted an analysis on posture congruency effects controlling for processing speed. Processing speed did predict congruency effects, *F*(1,52) = 5.80, *p* = 0.02, *η*^2^_*p*_ = 0.10, but there was no interaction with age group, *F*(1,52) = 0.73, *p* = 0.40, *η*^2^_*p*_ = 0.01. There was also no three-way interaction with angle of rotation, *F*(2,104) = 0.04, *p* = 0.96, *η*^2^_*p*_ < 0.001. Therefore, apart from slower response latency in general, older adults showed a similar pattern of responding to younger adults.

##### Perspective-tracking (Level 1 VPT) with stimulation in older adults

Bodily position had no effect on response times (*F*_(1, 54)_ = 0.75, *p* = 0.391, *η*^2^ ≤ 0.001). Also, all other main effects and interaction effects were nonsignificant: ANGLE (*F*_(2, 108)_ = 2.15, *p* = 0.121, *η*^2^ = 0.001), STIM TYPE (*F*_(1,54)_ = 0.39, *p* = 0.534, *η*^2^ = 0.001), STIM SITE (*F*_(1,54)_ = 1.86, *p* = 0.179, *η*^2^ = 0.023), STIM TYPE × POSTURE × ANGLE × STIM SITE (*F*_(2, 108)_ = 0.50, *p* = 0.605, *η*^2^ ≤ 0.001), STIM TYPE × POSTURE × ANGLE (*F*_(2, 108)_ = 0.95, *p* = 0.388, *η*^2^ ≤ 0.001), STIM TYPE × ANGLE (*F*_(2, 108)_ = 0.20, *p* = 0.818, *η*^2^ ≤ 0.001), STIM TYPE × posture (*F*_(1,54)_ = 0.51, *p* = 0.477, *η*^2^ ≤ 0.001), STIM TYPE × STIM SITE (*F*_(1,54)_ = 0.62, *p* = 0.436, *η*^2^ = 0.002). Therefore, tDCS to either stimulation site did not affect perspective-tracking.

##### Perspective-taking (Level 2 VPT) with stimulation in older adults

As expected, body posture had a significant effect on response times (*F*_(1, 55)_ = 5956.71, *p* < 0.001, *η*^2^ = 0.016), with slower responses when the participant’s bodily position was incongruent with the location of the avatar (*t*(55) = − 7.53, *p*_Tukey_ < 0.001). Further, we identified an effect for angle of rotation (*F*_(2,110)_ = 36.32, *p* < 0.001, *η*^2^ = 0.031), with post hoc tests showing response times increasing with greater angular disparity between participant and avatar (participants reacted faster at 60° compared to 160°, *t*(55) = − 6.02, *p*_Tukey_ < 0.001, and also at 110° compared to 160°,* t*(55) = − 10.26, *p*_Tukey_ < 0.001). Yet, there was no interaction between ANGLE × posture (*F*_(2,110)_ = 1.46, *p* = 0.237, *η*^2^ ≤ 0.001), indicating that a posture congruency effect was observed at all angular disparities, replicating previous reports (e.g. Kessler & Rutherford, 2010; Kessler et al., 2014, Martin et al., 2020).

These results were also found in the study by Martin et al. (2020) in younger adults. However, contrary to the results by Martin et al. (2020), we could not find any significant stimulation effects in our sample of older adults: STIM TYPE (*F*_(1,55)_ = 0.25, *p* = 0.621, *η*^2^ = 0.001), STIM SITE (*F*_(1,55)_ = 0.27, *p* = 0.603, *η*^2^ = 0.004), STIM TYPE × POSTUREX ANGLE × STIM SITE (*F*_(2, 110)_ = 0.43, *p* = 0.652, *η*^2^ ≤ 0.001), STIM TYPE × POSTURE × ANGLE (*F*_(2, 110)_ = 1.13, *p* = 0.327, *η*^2^ ≤ 0.001), STIM TYPE × ANGLE (*F*_(2, 110)_ = 0.67, *p* = 0.515, *η*^2^ ≤ 0.001), STIM TYPE × POSTURE (*F*_(1,55)_ = 0.11, *p* = 0.747, *η*^2^ = < 0.001), STIM TYPE x STIM SITE (*F*_(1,55)_ = 3.02, *p* = 0.088, *η*^2^ = 0.006). Therefore, tDCS to either stimulation site did not affect perspective-taking in our sample of older adults.

### Differences in field intensity

**Fig. 3:**
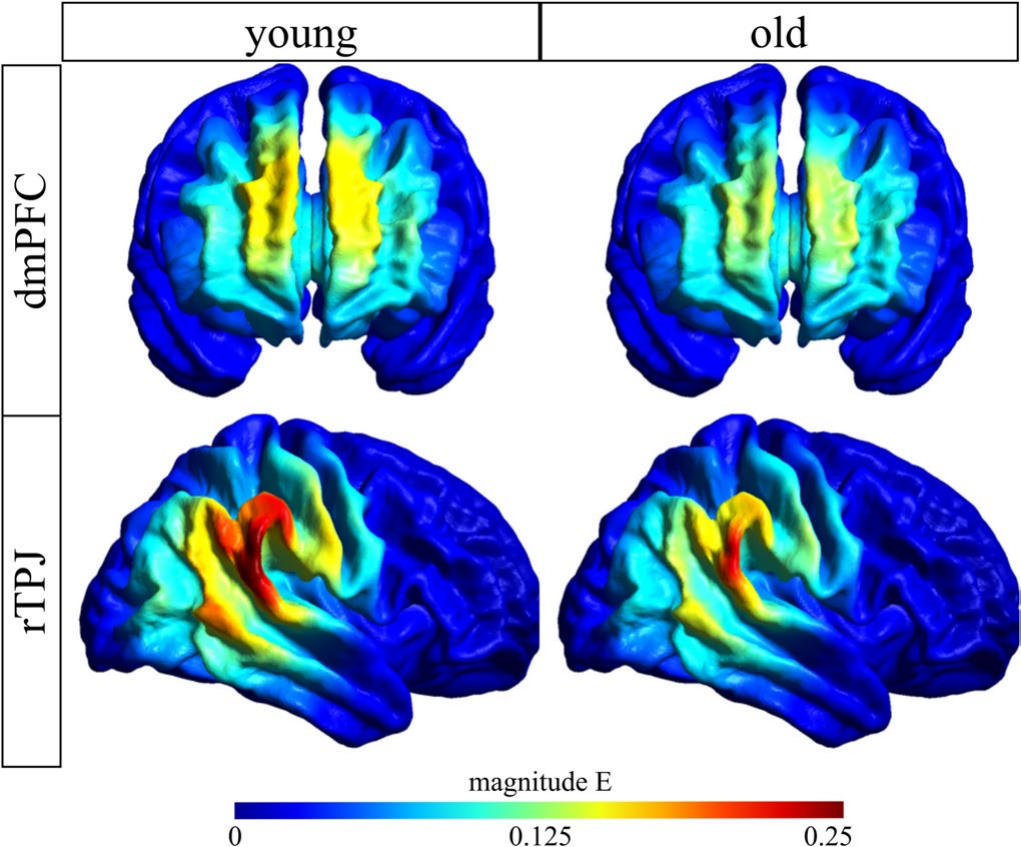
Overview of simulated current distribution induced by the two montages (dmPFC, rTPJ), separately for both age groups

Figure 3 provides an overview of simulated current distribution induced by the two montages (dmPFC, rTPJ), separately for both age groups. Visual inspection of group averages demonstrates focal current delivery, with peak intensity at and around the target regions, with overall reduced current intensity in older adults.

**Fig. 4:**
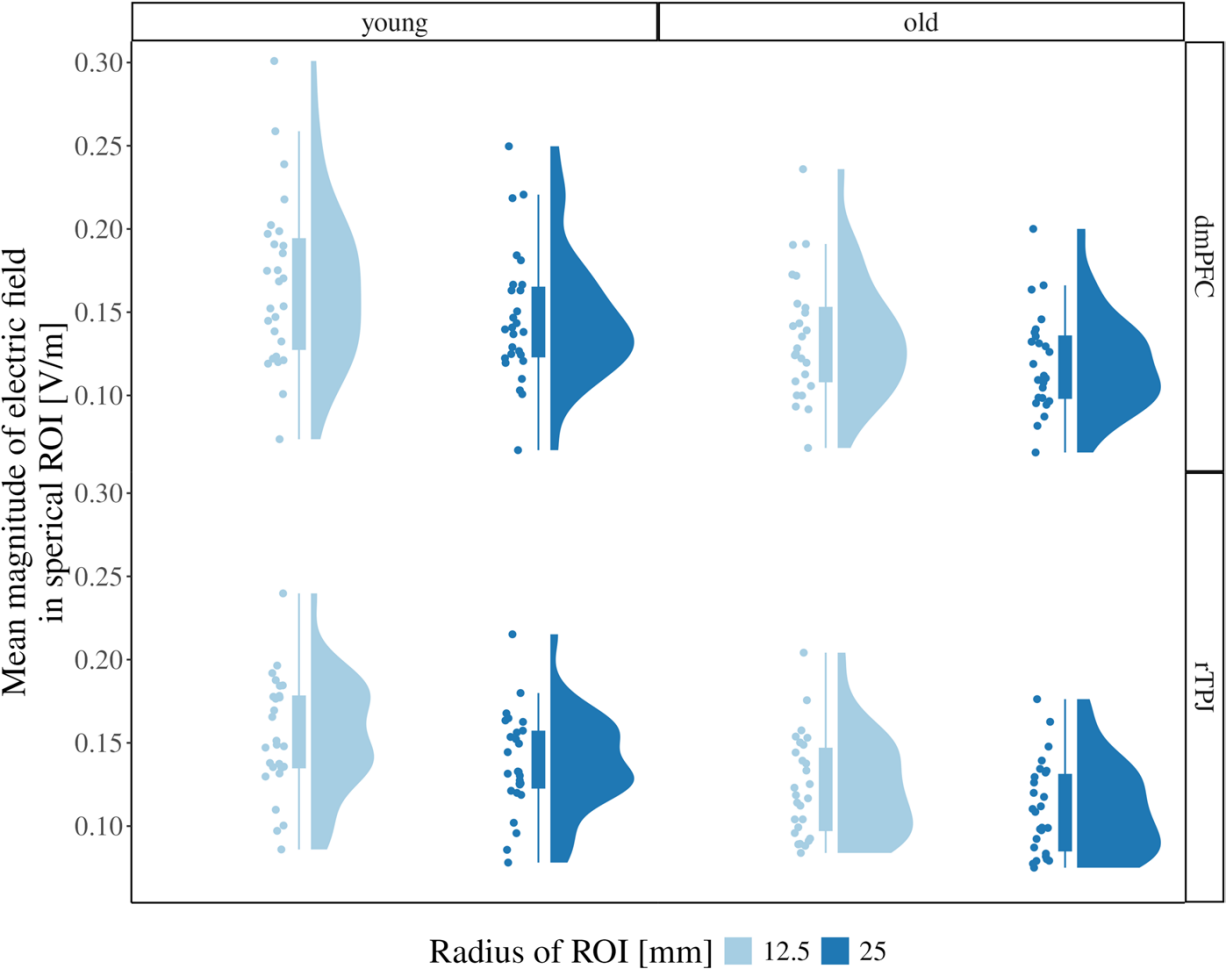
ROI analysis

This was subsequently confirmed by the ROI analyses (Fig. 4). Age group showed a significant effect for rTPJ (***F***_(1, 52)_ = 11.95, *p* = 0.0011, *η*^2^ = 0.18) and dmPFC tDCS (***F***_(1, 50)_ = 7.251, *p* = 0.00962, *η*^2^ = 0.12), indicating that current strength in the target regions for tDCS was reduced in older compared to younger individuals. Furthermore, there was a significant effect of ROI radius for rTPJ (***F***_(1, 52)_ = 334.192, *p* < 0.001, *η*^2^ = 0.05) and dmPFC (***F***_(1, 50)_ = 103.212, *p* < 0.001, *η*^2^ = 0.04), with higher induced field strengths in the smaller compared to the larger ROI. This confirmed focal current delivery by our setup. There was no significant interaction between age group and radius for the rTPJ (***F***_(1, 52)_ = 2.933, *p* = 0.0928, *η*^2^ < 0.001) or dmPFC (***F***_(1, 50)_ = 2.069, *p* = 0.157, *η*^2^ < 0.001), suggesting comparable focality in both age groups.

### Adverse events of stimulation

Forty-one mild adverse events were reported by the older participants during active stimulation, and 35 mild adverse events were self-reported by participants during sham stimulation (see also Table 3). No serious adverse events were reported, and no participant terminated participation due to occurrence of adverse events.

**Table 3:** Adverse events of stimulation

	Total *N* = 61	Active stimulation *N* = 59	Sham stimulation *N* = 60
Total number of adverse events	75	41	34
Itching	11	6	5
Pain	1	1	0
Burning	17	8	9
Warmth/heat	14	8	6
Metallic/iron taste	0	0	0
Fatigue/decreased alertness	9	6	3
Other	23	12	11

Reported values are absolute frequency of the respective adverse events

### Positive and negative mood

Analyses revealed no significant differences between the positive pre- and post-scores (*t*[27] = − 0.57, *p* = 0.571; *t*[27] = − 0.41, *p* = 0.687) nor the negative pre-and post-scores (*t*[27] = − 0.38, *p* = 0.707; *t*[27] = − 0.15, *p* = 0.879) for the dmPFC tDCS group between the anodal and the sham group. For the rTPJ group, there were also no significant differences between the positive pre- and post-scores (*t*[26] = − 0.06, *p* = 0.949; *t*[27] = − 0.39, *p* = 0.699) nor the negative pre-and post-scores (*t*[26] = − 0.28, *p* = 0.782; *t*[27] = − 0.53, *p* = 0.603).

## Discussion

The present study investigated potential differences in embodied visual perspective taking and tracking between healthy young and older adults. In addition, we explored if perspective taking can be modulated by rTPJ tDCS in a regionally and task specific way, similar to previous results observed in younger adults (Martin et al., 2020). For this purpose, we conducted a sham-controlled, double-blind, cross-over study that included two randomized experimental groups (healthy older adults, receiving either dmPFC or rTPJ tDCS), and a healthy young control group for comparison of behavioral outcomes (without stimulation). Our main findings are that despite an overall slowing, older adults are as embodied as young individuals. However, tDCS had no effect on embodied perspective taking, which may be related to either reduced current delivery to the target regions due to age-associated changes in skull or brain anatomy or functional reorganization of the networks supporting socio-cognitive processes related to VPT.

Across both groups we show results that are generally consistent with the theory that two distinct cognitive processes are relevant for perspective tracking and perspective taking [26]. During level two perspective taking, the embodied factors of angle of rotation and body posture significantly affected response times. Specifically, the greater the rotation requirements, the slower the response, suggesting an embodied rotation to align the egocentric perspective with that of the avatar. Moreover, slower responses were observed when the participants’ posture was incongruent to that of the avatar, providing evidence for body posture as an embodied component relevant to perspective taking. Despite the aging process providing an excellent model for studying embodied factors due to physical and cognitive changes across the lifespan, to date, little research has been conducted in this domain. It has been suggested that cognition in older adults is less embodied, with older adults being more reliant on visual processing [11]. For example, during a mental rotation task with three different imagery types: an alphanumeric symbol, a two-dimensional image of a hand, or a three-dimensional whole-body image, older adults showed the greatest difficulty in the whole-body condition [34]. Further research has shown that older adults find tasks requiring an egocentric rotation strategy especially difficult [35, 36]. However, we show no age-related difference on either embodied aspect of level two perspective taking suggesting that embodied processes may remain a viable strategy independent of age-related bodily changes. In the perspective tracking task, we also found no evidence for a contribution of embodied factors, consistent with previous research in younger adults [5]. As with level two perspective taking, the only age-related difference was general slowing in the older adults.

Despite the novel findings demonstrating maintained embodied factors related to level two perspective taking in older adults, we find no effect of anodal stimulation to the rTPJ on modulating postural effects. Therefore, we show that stimulation effects in younger adults can not readily be translated to healthy older adults in the domain of perspective taking. Previous research has found mixed results when comparing tDCS effects in young and older adults (see [37]), and we have previously shown that dissociable stimulation effects of rTPJ tDCS observed in younger adults were not found in healthy older adults [17]. A straightforward explanation for this effect, and potentially those in other cognitive domains, could be the reduced current delivery to both target brain regions in the older groups, which was particularly pronounced for the rTPJ. However, the required threshold for inducing physiologically meaningful changes in neural excitability is currently not known and may vary even within groups of young and older individuals (for review see [38]). Hence, the functional relevance of reduced current flow to the target regions needs to explored in the future, for example, by prospective computational modelling of current flow aimed at equalizing current intensity within and across participants.

However, several other factors may explain the discrepancy between results. A lack of consistent effects of social brain stimulation on perspective-taking has been demonstrated in a recent meta-analysis [16]. However, the meta-analysis did not consider embodied factors such as bodily posture used in the present study, and our previous study in younger adults that used the same experimental and stimulation set-up [5]. Baseline cognitive differences may also explain stimulation differences in young and older adults. Previous studies have identified age-related stimulation responses dependent on baseline cognitive functioning [37, 39]. As expected, older adults responded slower on both level one perspective tracking and level two perspective taking tasks. Processing speed has been shown to affect stimulation response in the clinical domain and may explain the lack of stimulation response in older adults in the present study. Processing speed is also associated with reduced functional connectivity within a broader right frontoparietal network [40], and brain stimulation response is associated with baseline functional connectivity between the stimulated and connected regions [41]. Future research is required to further understand the underlying neural and cognitive factors that predict stimulation response in older adults, including reorganization of functional brain networks supporting VPT.

In this context, the rTPJ is frequently identified to be activated across a wide range of cognitive tasks, including tasks such as spatial attention and a wide range of cognitive processes considered important for social [42, 43]. Relevant to VPT, the rTPJ has been implicated in self-other distinction [44], self-inhibition [45], own-body imagery [46], and embodiment [5, 6, 33]. The rTPJ is a key hub for many brain networks and can be parcellated according to connectivity profiles accordingly [47]. However, little is known about how this parcellation and the network-level properties of the rTPJ change across the lifespan. Aging is associated with dedifferentiation of neural and cognitive processes [48, 49], which likely reduce the modular specificity of key hub regions such as the rTPJ. This offers one potential reason for the age-specific effects of stimulation to the rTPJ observed in the present study and previously in different age-groups [17]. Our results also support the absence of a causal role for the dmPFC in level one perspective tracking or level two perspective taking, when there is no online cognitive control requirement to switch between egocentric and allocentric perspectives.

It is important to note that visual perspective tracking and -taking have been measured using different tasks [16, 50], and, the results of this study may not generalize to other tasks. However, by replicating the study used in [5], we further show that results obtained in young adults are not necessarily translatable to healthy older adults. Future studies are required to investigate the cognitive and neural predictors of stimulation response on VPT performance in older adults. Age-related changes are evident across the brain and alternate stimulation sites may modulate VPT performance in older adults. Focal stimulation may have reduced efficacy if small electrode placement errors are present [32]. As location of the rTPJ was only approximated using the 10–20 EEG system as a reference for our electrode placement at CP6, it is possible that heterogeneity of response site, coupled with underlying heterogeneity of brain structure, has affected the efficacy of stimulation response. This issue may be compounded in older adults due to further heterogeneity due to age-related brain change, both structurally and functionally. Future research is required to understand how underlying properties of the brain may mediate stimulation response in relation to visual perspective taking in older adults.

In conclusion, we provide the first evidence for maintained embodied processing relevant to level two perspective taking in older adults, challenging theoretical accounts of reduced embodied cognition in older adults. We also show that despite similar embodied effects, focal stimulation to the rTPJ previously identified in younger adults to modulate embodied factors had no effect in older adults.

## Data Availability

The data that support the findings of this study are available from the corresponding author, upon reasonable request.
